# Sea Spectral Estimation Using ARMA Models

**DOI:** 10.3390/s21134280

**Published:** 2021-06-23

**Authors:** Marta Berardengo, Giovanni Battista Rossi, Francesco Crenna

**Affiliations:** Department of Mechanical, Energy, Management and Transportation Engineering, University of Genova, Via Opera Pia 15A, 16145 Genova, Italy; g.b.rossi@unige.it (G.B.R.); francesco.crenna@unige.it (F.C.)

**Keywords:** sea waves monitoring, ARMA model, Prony method, spectral estimation

## Abstract

This paper deals with the spectral estimation of sea wave elevation time series by means of ARMA models. To start, the procedure to estimate the ARMA coefficients, based on the use of the Prony’s method applied to the auto-covariance series, is presented. Afterwards, an analysis on how the parameters involved in the ARMA reconstruction procedure—for example, the signal time length, the number of poles and data used—affect the spectral estimates is carried out, providing evidence on their effect on the accuracy of results. This allowed us to provide guidelines on how to set these parameters in order to make the ARMA model as accurate as possible. The paper focuses on mono-modal sea states. Nevertheless, examples also related to bi-modal sea states are discussed.

## 1. Introduction

Knowledge of the weather conditions in the context of marine engineering applications is often a key point for structural safety, and also for people’s comfort in the case of ships in navigation. In this context, it is very important to have a reliable estimation of the sea spectrum because it has a significant influence on ship motion [1,2], but also on the forces to which ships and offshore structures are subject [3]. Moreover, it constitutes the input of the procedures for the estimation of the sea parameters which are then used for several purposes such as fatigue lifetime estimation [3], or to avoid ship dynamic instabilities [4]. Consequently, different approaches were proposed in the literature to estimate the sea features, starting from either onboard measurements (e.g., ship motion [5,6,7,8,9]) or time signals of wave level. Taking into consideration the latter approach, on the one hand, it is possible to successfully use non-parametric methods such as the Welch’s [10] and Thomson’s [11] methods for estimating sea spectrum [12,13,14]. On the other hand, parametric methods could also be employed, with the consequent advantage of obtaining a model of the sea spectrum, which can be then used for advanced predictions of sea actions on ships and structures. The availability of a model able to predict the sea state is essential not only in the fields of navigation, ship safety and for the estimation of the forces acting on offshore structures, but also, as an example, in the field of the wave energy converters, where the prediction of the wave elevation allows for the optimization of the control action, leading in turn to the possibility of maximizing the power extraction [15].

Among parametric methods, here the use of Auto-Regressive Moving Average (ARMA) models is taken into consideration because it was already evidenced in the literature that it can be fruitfully employed for output-only identification processes in both sea spectrum estimation and, more generally, in structural dynamics [16].

Considering the specific problem related to sea wave spectrum estimation, Spanos [17] and Mandal et al. [18] showed that ARMA models can be successfully applied and provided examples, mainly related to mono-modal sea state, of their application. Nevertheless, some points are still missing in the literature about the use of ARMA models in sea spectral reconstruction. First of all, since the Auto-regressive (AR) and Moving Average (MA) parts of the ARMA model are usually estimated with a two-step procedure, also involving numerical minimisations, a statistical analysis of the results that can be obtained is important for comprehending the actual potentiality of the ARMA approach in the context of sea estimation. Currently, such an analysis is missing. This in turn implies that no specific analysis can be found related to the influence of the different input parameters, set by the user at the beginning of the identification procedure, on the differences between the reference and the reconstructed sea power spectral estimate. Indeed, one of the main drawbacks related to the use of such models is related to the fact that their estimation procedure requires the setting of some free parameters that can significantly affect the quality of the estimated model. In the literature, no clear indications are given about how to choose the values of these input parameters and only generic indications are provided. In this context, this paper will address some of these points in order to clarify strengths and drawbacks of the considered identification approach, also providing guidelines for setting some of the key parameters of the procedure such as the number of samples that can be used, given a certain time series, and the number of the poles of the ARMA model, which are shown to strongly affect the effectiveness of the power spectral density reconstruction.

To this purpose, the paper is organised into nine sections where Section 2, Section 3 and Section 4 are related to the methods on which the proposed estimation procedure is based, Section 5 describes the method used to simulate the data for testing this approach, while Section 6, Section 7 and Section 8 are aimed at presenting the results and at their discussion. In particular, the paper briefly recalls in Section 2 the model assumed for the description of the sea wave elevation time series, which is here considered as a stochastic process, and provides some useful mathematical relations needed for the subsequent estimates of the model parameters. The estimation procedure is then described in Section 3; it combines different established techniques such as the Prony’s and the Shanks’ method, applied to the auto-covariance of the time series and used together to achieve the best results in terms of parameter accuracy. Despite that the used techniques are not new, they are recalled in Section 3 to summarize the whole estimation procedure and to let the reader understand the reasons behind the choice of the selected algorithms and the subsequent analysis of Section 4, related to the choice of the parameters involved in the estimation procedure. Section 5 explains how the data of the sea wave elevation and the sea power spectral density are simulated for given sea state conditions. Then, Section 6 proposes an analysis of the effect of the parameters involved in the proposed estimation procedure on the estimate accuracy. Particularly, the attention is focused on the effect of the number of auto-covariance samples used for the estimation process on the accuracy of the results, also in relation to the whole length of the time series, on the effect of the initial number of poles selected for the ARMA model and on how to improve the reliability of the estimates in the case of short-time series. All the analyses presented in Section 6 are related mono-modal sea states, where only the wind contribution is considered. Section 7 applies instead the procedure to different mono-modal sea states and to a case of bi-modal sea where both the wind and swell contributions are present. Despite that no detailed analysis is proposed here about this complex sea condition, the method is shown to be promising and worthy of further investigation. Finally, Section 8 provides guidelines on how to set the main parameters of the procedure in order to maximise the accuracy of the spectral reconstruction.

## 2. ARMA Modelling of the Wave Elevation Time Series

The wave elevation time series x(n) is considered as a stationary real valued (as all the other time signals considered in the following), zero-mean stochastic process. For the Wold’s decomposition theorem, any discrete stationary stochastic process can be expressed as the sum of two uncorrelated processes: one deterministic and another stochastic. Therefore, it is possible to model the time series x(n) as a process generated by the filtering of white noise w(n) with a Linear-Shift-Invariant (LSI) filter, having unit sample response h(n). In the considered case, the LSI filter in the z-domain is represented as a stable, causal rational function with *p* poles and *q* zeros (with p≥q):

(1) $$H\left(z\right)=\frac{B_{q}\left(z\right)}{A_{p}\left(z\right)}=\frac{\sum_{i=0}^{q}b_{i}z^{-i}}{1+\sum_{i=1}^{p}a_{i}z^{-i}}=\frac{\sum_{i=0}^{q}b_{i}z^{-i}}{\prod_{i=1}^{p}\left(1-\lambda_{i}z^{-i}\right)}$$

where H(z) is the z-transform of h(n) and $\lambda_{i}$ are the poles of the LSI system and are the solutions of the polynomial equation $1+\sum_{i=1}^{p}a_{i}z^{-i}=0$.

The random processes x(n) and w(n) are thus linked by the following linear difference equation with constant coefficients:

(2) $$x\left(n\right)=-\overset{p}{\underset{i=1}{\sum }}a_{i}x\left(n-i\right)+\overset{q}{\underset{i=0}{\sum }}b_{i}w\left(n-i\right)$$

where the first summation represents the AR and the second the MA parts of the ARMA model assumed to describe the stochastic process x(n).

Given the input-output relation between x(n) and w(n), the time series x(n) can be expressed as the convolution between the driving input w(n) and the unit sample response h(n):

(3) $$x\left(n\right)=w\left(n\right)⊛h\left(n\right)=\overset{\infty }{\underset{i=-\infty }{\sum }}h\left(i\right)w\left(n-i\right)$$

where ⊛ is the convolution operator.

The cross-covariance between the input and output can be then expressed as:

(4) $$r_{\mathrm{xw}}\left(k\right)=E\left\{w\left(n\right)x\left(n+k\right)\right\}=E\left\{\overset{\infty }{\underset{i=-\infty }{\sum }}h\left(i\right)w\left(n-i+k\right)w\left(n\right)\right\}=\overset{\infty }{\underset{i=-\infty }{\sum }}h\left(i\right)r_{w}\left(k-i\right)=h\left(k\right)⊛r_{w}\left(k\right)$$

where E{·} indicates the expected value, w(n) is assumed wide sense stationary and $r_{w}$ is the auto-covariance of the input. Analogously, the cross-covariance $r_{\mathrm{wx}}$ can be derived as:

(5) $$r_{\mathrm{wx}}\left(k\right)=h\left(-k\right)⊛r_{w}\left(k\right)$$

Finally, using Equations (3)–(5), the auto-covariance of the output results:

(6) $$r_{x}\left(k\right)=E\left\{x\left(n\right)x\left(n+k\right)\right\}=E\left\{x\left(n+k\right)\overset{\infty }{\underset{i=-\infty }{\sum }}h\left(i\right)w\left(n-i\right)\right\}=\overset{\infty }{\underset{i=-\infty }{\sum }}h\left(i\right)r_{\mathrm{xw}}\left(k+i\right)=r_{w}\left(k\right)⊛h\left(k\right)⊛h\left(-k\right)$$

Noting that h(k)⊛h(−k) can be also seen as the auto-covariance of the unit sample response h(n) and that the auto-covariance of the driving input can be expressed as the variance $\sigma_{w}^{2}$ of the stochastic process w(n) multiplied by the unit sample [19], the auto-covariance of the process x(n) of Equation (6) can be also expressed as a scaled version of the auto-covariance, $r_{h}\left(n\right)$, of h(n):

(7) $$r_{x}\left(k\right)=\sigma_{w}^{2}r_{h}\left(k\right)$$

The power spectral estimate $S_{x}$ of x(n) can be finally obtained from the z-transform of Equation (7), that can be expressed, using Equation (1), as:

(8) $$S_{x}\left(z\right)=\sigma_{w}^{2}H\left(z\right)H\left(z^{-1}\right)=\sigma_{w}^{2}\frac{B_{q}\left(z\right)B_{q}\left(z^{-1}\right)}{A_{p}\left(z\right)A_{p}\left(z^{-1}\right)}$$

## 3. ARMA Model Estimation Method

With the aim of estimating the model parameters to have a spectral reconstruction of the wave signal, it is convenient to manipulate Equation (2), multiplying both sides by x(n−k) and taking the expected values:

(9) $$E\left\{x\left(n\right)x\left(n-k\right)\right\}+\overset{p}{\underset{i=1}{\sum }}a_{i}E\left\{x\left(n-i\right)x\left(n-k\right)\right\}=\overset{q}{\underset{i=0}{\sum }}b_{i}E\left\{w\left(n-i\right)x\left(n-k\right)\right\}$$

using Equation (5) and recalling that h(n) is assumed to be causal (i.e., h(n)=0 for n<0), Equation (9) can be reduced to:

(10) $$r_{x}\left(k\right)+\overset{p}{\underset{i=1}{\sum }}a_{i}r_{x}\left(k-i\right)=\sigma_{w}^{2}\overset{q}{\underset{i=k}{\sum }}b_{i}h\left(i-k\right)$$

Then, calling $c_{q}\left(k\right)=\overset{q-k}{\underset{i=0}{\sum }}b_{i+k}h\left(i\right)$ and noting that the right-hand side of Equation (9) is equal to zero for k>q, Equation (10) becomes:

(11) $$r_{x}\left(k\right)+\overset{p}{\underset{i=1}{\sum }}a_{i}r_{x}\left(k-i\right)=\{\begin{array}{cc} \sigma_{w}^{2}c_{q}\left(k\right) & 0\leq k\leq q\\ 0 & k>q \end{array}$$

Equation (11) are the Yule Walker equations of the ARMA process of Equation (2). Looking at their expression, it is possible to notice that the second set of equations (i.e., those for k>q) can be used to solve the linear problem related to the estimation of the AR coefficients $a_{i}$. This procedure is referred to as the Modified Yule Walker Method (MYW) [20] and uses the unbiased sample correlation coefficients as estimates of the auto-covariance $r_{x}$:

(12) $$r_{x}\left(k\right)≃\frac{1}{N-k}\overset{N}{\underset{i=k+1}{\sum }}x_{i}x_{i-k}$$

where N is the total number of samples of x(n). However, in order to find the best fit of the AR parameters in the least square sense and to improve the MYW estimates, one of the best procedures is to apply the Prony’s method to the auto-covariance function of the stochastic process x(n) [21,22,23].

As for the MA parameters, a better approach with respect to Prony’s method is that suggested by Shanks [24], which allows finding the MA coefficients in a least square sense as well. Therefore, a two-step procedure is herein followed to find the ARMA parameters.

### 3.1. Prony Based Estimation of the AR Parameters

Equation (7) expresses the auto-covariance of the stochastic process x(n) as a scaled version of the auto-covariance of the unit sample response h(n). Since h(n) is a causal sequence that can be expressed as a sum of non-increasing exponentials, the causal part of its auto-covariance function can also be expressed as a sum of damped exponentials with the same set of poles [25,26]. Therefore, recalling Equations (1) and (7), it is possible to write:

(13) $$r_{x}\left(k\right)=\overset{p}{\underset{i=1}{\sum }}\alpha_{i}\exp\left(\lambda_{i}k\right)\;\;\;\;\;\;k>q$$

Recalling that the model of the time series assumed by the Prony’s method [27] has the same form of the auto-covariance of Equation (13), it is possible to estimate the AR coefficients by deriving the poles $\lambda_{i}$ applying the Prony’s method to the auto-covariance sequence $r_{x}$ [22] and solving for β, with a least square approach, the following problem:

(14) $$\left[R_{x}\right]\beta =-{\tilde{R}}_{x}$$

where:

(15) $$\left[R_{x}\right]=\left[\begin{array}{cccc} r_{x}\left(1\right) & r_{x}\left(2\right) & \cdots & r_{x}\left(p\right)\\ r_{x}\left(2\right) & r_{x}\left(3\right) & \cdots & r_{x}\left(p+1\right)\\ \vdots & \vdots & \ddots & \vdots \\ r_{x}\left(k_{0}\right) & r_{x}\left(k_{0}+1\right) & \cdots & r_{x}\left(p+k_{0}\right) \end{array}\;\right]$$

(16) $${\tilde{R}}_{x}=\left[\begin{array}{c} r_{x}\left(p+1\right)\\ \vdots \\ r_{x}\left(p+k_{0}+1\right) \end{array}\;\right],\;\;\beta =\left[\begin{array}{c} \beta_{0}\\ \vdots \\ \beta_{p-1} \end{array}\;\right]$$

where $k_{0}$ is an integer equal to or higher than p (usually, $k_{0}>p$ to have more equations than unknowns) and $\beta_{i}$ are the coefficients of the polynomial equations having the roots equal to

(17) $$\beta_{0}+\beta_{1}\lambda +\beta_{2}\lambda^{2}+\ldots +\beta_{p}\lambda^{p}=0,\;\;\;\mathrm{with}\;\;\beta_{p}=1$$

It is noticed that, sometimes, the auto-covariance for k=0 is not used in Equation (15) (as done here) due to the possible contribution of additional noise to the signal [28]. It is also noticed that the number of auto-covariance samples used to write Equation (14) will be referred to as φ from here on, where $\varphi =p+k_{0}+1\geq 2p+1$.

Finally, the ARMA poles $\lambda_{i}$ can be found solving Equation (14) for β and then solving Equation (17), obtaining the roots of the polynomial equation. The AR coefficients can be then estimated using Equation (1) and the $\alpha_{i}$ values can be found using Equation (13).

### 3.2. MA Coefficients and Signal Spectral Estimation

The Shanks’ method for the MA coefficient estimation is based on the description of the one-sided auto-covariance sequence $r_{x}$ as the unit sample response of a linear system $H_{r}\left(z\right)$, whose poles are those of the ARMA model (see Equation (13)):

(18) $$H_{r}\left(z\right)=\frac{N_{q}\left(z\right)}{A_{p}\left(z\right)}$$

where $N_{q}\left(z\right)=n_{1}z^{-1}+n_{2}z^{-2}+\ldots +n_{q}z^{-q}$.

Therefore, with the aim of estimating the numerator coefficients of Equation (18), it is possible to minimize the error e(n) between the auto-covariance $r_{x}\left(k\right)$ and its estimate ${\hat{r}}_{x}\left(k\right)$ obtained using Equation (18):

(19) $$e\left(k\right)=r_{x}\left(k\right)-{\hat{r}}_{x}\left(k\right)=r_{x}\left(k\right)-\overset{q}{\underset{i=1}{\sum }}n_{i}h_{\mathrm{ra}}\left(k-i\right)$$

where $h_{\mathrm{ra}}$ is the impulse response of the function $1/A_{p}\left(z\right)$.

The problem thus reduces to the least square solution with respect to n of the problem $\left[h_{r}\right]n=r_{0}$:

(20) $$\left[\begin{array}{cccc} h_{\mathrm{ra}}\left(0\right) & 0 & \cdots & 0\\ h_{\mathrm{ra}}\left(1\right) & h_{\mathrm{ra}}\left(0\right) & \cdots & 0\\ \vdots & \vdots & \ddots & \vdots \\ h_{\mathrm{ra}}\left(\varphi -1\right) & h_{\mathrm{ra}}\left(\varphi -2\right) & \cdots & h_{\mathrm{ra}}\left(\varphi -q\right) \end{array}\;\right]\left[\begin{array}{c} n_{1}\\ \vdots \\ n_{q} \end{array}\;\right]=\left[\begin{array}{c} r_{x}\left(1\right)\\ r_{x}\left(2\right)\\ \vdots \\ r_{x}\left(\varphi \right) \end{array}\;\right]$$

Once that $N_{q}\left(z\right)$ is derived, the power spectral estimate of x(n) can be expressed, using Equation (18), as:

(21) $$S_{x}\left(z\right)=\frac{N_{q}\left(z\right)}{A_{p}\left(z\right)}+r_{x}\left(0\right)+\frac{N_{q}\left(z^{-1}\right)}{A_{p}\left(z^{-1}\right)}=\frac{N_{q}\left(z\right)A_{p}\left(z^{-1}\right)+r_{x}\left(0\right)A_{p}\left(z\right)A_{p}\left(z^{-1}\right)+N_{q}\left(z^{-1}\right)A_{p}\left(z\right)}{A_{p}\left(z\right)A_{p}\left(z^{-1}\right)}$$

Finally, the MA coefficients can be estimated using Equations (8) and (21).

## 4. Setting the ARMA Estimation Procedure: The Free Parameter Selection

Section 2 explained how the stochastic process related to the sea wave elevation can be described by an ARMA model and Section 3 proposed a procedure, based on the auto-covariance estimates of the time series, to derive the parameters of the model and thus allowing us to derive, using Equation (21), the sea spectral estimates. However, to apply the ARMA estimation procedure, the values of some free parameters have to be defined since they affect the quality of the spectral estimates. Therefore, the involved free parameters have to be properly set, as described in Section 4.1 and Section 4.2.

### 4.1. AR Order Selection

The first problem to be addressed is related to the choice of the unknown p order of the AR model of the ARMA process. Several studies show that increasing the AR order improves the effectiveness of the various available algorithms [29,30]. Moreover, Friedlander and Porat [31] showed that, since the AR problem (i.e., that related to the AR part of the ARMA model) can be seen as a least square predictor of an AR process on the auto-covariance sequence, increasing the p order leads to more accurate estimates and that the poles of the actual AR model are a subset of those of the higher order predictor $\hat{p}$.

In order to set the value of the increased order $\hat{p}$ of the AR problem, several criteria can be used. Among them, the well-known Akaike Information Criterion [32] (AIC) is used here, where the order is chosen as the value which minimizes the following expression [31,33]:

(22) $$\mathrm{AIC}=2p+\varphi \ln\frac{e_{r}}{\varphi -p}$$

where $e_{r}=\sum_{k=1}^{\varphi }{\left[r_{x}\left(k\right)-\sum_{i=1}^{p}\alpha_{i}\exp\left(\lambda_{i}k\right)\right]}^{2}$.

Once the increased-order AR model is derived using the procedure of Section 3.1, only the poles that fit the main signal components have to be selected, discarding those mainly related to the fit of the noise. This procedure can be carried out following an energetic criterion. Knowing the poles $\lambda_{i}$, it is possible to factorise the z-transform of Equation (13) into first, $L_{i}\left(z\right)$, and second, $M_{i}\left(z\right)$, order contributions:

(23) $$L_{i}=\frac{F_{1,i}z^{-1}}{1+f_{1,i}z^{-1}},\;\;\;M_{i}=\frac{S_{1,i}z^{-1}+S_{2,i}z^{-2}}{1+s_{1,i}z^{-1}+s_{2,i}z^{-2}\;}$$

If $L_{i}\left(z\right)$ or $M_{i}\left(z\right)$ are found to be unstable, they can be stabilized by reflecting the unstable roots into the unit circle [34].

Then, the energy associated to each system is calculated as:

(24) $$e_{f_{i}}=\frac{1}{2\pi j}\oint z^{-1}{\left|\frac{F_{1,i}z^{-1}}{1+f_{1,i}z^{-1}}\right|}^{2}dz,\;e_{s_{i}}=\frac{1}{2\pi j}\oint z^{-1}{\left|\frac{S_{1,i}z^{-1}+S_{2,i}z^{-2}}{1+s_{1,i}z^{-1}+s_{2,i}z^{-2}\;}\right|}^{2}dz$$

Finally, only the systems with the highest energy are considered (i.e., those whose energy is higher than a fixed threshold level $t_{r}$, see Section 8).

### 4.2. Number of Equations of the Least-Square Problems

The second parameter that has to be defined is the number of equations used to solve the minimisation problems. Indeed, the estimates of the ARMA parameters are based on the least square solution of the two problems of Equations (14) and (20). Therefore, the value of the auto-covariance samples used in the procedure, φ, constrains the number of equations used to define the problems which in turn affects the accuracy of the results. Particularly, an increase of the equations is expected to improve the quality of the results. However, it is noticed that since the problem involves the auto-covariance estimates obtained using Equation (12), φ cannot be increased indefinitely. Indeed, for large lags k, the variance of the auto-covariance estimates increases also due to a decrease of the denominator N−k in Equation (12) [19,35,36], leading to an annulment of the beneficial effect of the increase of the number of equations.

Further details about how to choose the best φ value are given in Section 6.

## 5. Case-Studies: Wave Elevation Time Series Generation and Spectral Description

The algorithm described in Section 3 for the spectral estimation of the sea wave time series, based on an ARMA model of the phenomenon, has been tested on simulated signals representing the sea wave elevation in different sea conditions. To generate these signals, the reference sea power spectral density (PSD) has been defined at first and then used to generate the signal time histories x(n).

In general, the sea state can be described by a combination of wind sea and uncorrelated swell and its PSD, S(ω), can be obtained joining the two contributions [37,38]:

(25) $$S\left(\omega \right)=S_{\mathrm{wind}}\left(\omega \right)+S_{\mathrm{swell}}\left(\omega \right)$$

where ω is the circular frequency and the spectral wind, $S_{\mathrm{wind}}\left(\omega \right)$, and swell, $S_{\mathrm{swell}}\left(\omega \right)$, components can be modelled by the JONSWAP spectrum $S_{J}\left(\omega \right)$ [37]:

(26) $$S_{J}\left(\omega \right)=A_{\gamma }S_{\mathrm{PM}}\left(\omega \right)\gamma^{\exp\left(-0.5{\left(\frac{\omega -\omega_{p}}{\sigma \omega_{p}}\right)}^{2}\right)}$$

In Equation (26) γ is the peak enhancement factor and $A_{\gamma }$ is the normalizing factor expressed as $A_{\gamma }=1-0.287\ln\left(\gamma \right)$. Given the wave peak period $T_{p}$, $\omega_{p}=2\pi /T_{p}$ is the peak frequency and σ is the spectral width parameter and is equal to 0.07 if $\omega \leq \omega_{p}$, 0.09 otherwise. In the event that no information about $T_{p}$ is available, it can be expressed, in the range of γ between 1 and 7, as a function of the wave mean period $T_{m}$ [37]:

(27) $$\frac{T_{m}}{T_{p}}=C+D\gamma +c\gamma^{2}+d\gamma^{3}$$

with C = 0.7303, D = 0.04936, c= −0.006556 and d = 0.000361.

Finally, $S_{\mathrm{PM}}$ in Equation (26) is the Pierson–Moskowitz spectrum [39]:

(28) $$S_{\mathrm{PM}}\left(\omega \right)=\frac{5}{16}H_{s}^{2}\omega_{p}^{4}\omega^{-5}\exp\left(-\frac{5}{4}{\left(\frac{\omega }{\omega_{p}}\right)}^{-4}\right)$$

where $H_{s}$ is the significant wave height.

Given the sea PSD, S(ω), the random wave elevation time series x(n) can be derived as the superposition of M equally spaced harmonic components at frequencies $\omega_{i}=i\Delta \omega$, with random phase $\varphi_{i}$ [40]:

(29) $$x\left(n\right)=\overset{M}{\underset{i=1}{\sum }}\sqrt{2S\left(\omega_{i}\right)\Delta \omega_{i}}\cos\left(\omega_{i}n\Delta_{t}+\varphi_{i}\right)$$

where $\Delta_{t}$ is the sampling time, defined as the inverse of the sampling frequency $f_{samp}$ expressed in Hertz (i.e., $\Delta_{t}=1/f_{samp}$).

The data used in the following analyses are mainly related to sea state conditions that can be described by single peak wave spectra related to sea wind. Nevertheless, the method for the sea PSD reconstruction has been applied also to double peak wave spectra due to both wind and swell to show the effectiveness and reliability of the procedure. The simulated sea conditions were chosen to represent different sea states from slight to rough, mono and bi-modal. In the following sections the analyses will be carried out on three case-studies. The values of the parameters used for the simulations are gathered in Table 1. Further information about the sampling frequency, the frequency resolution, the length of the time series and the value of the added noise will be specified each time in the next sections since they are varied in the different simulations to show their effect on the accuracy of the spectral estimates.

## 6. Sea Spectral Estimation: Results and Discussion

In the estimation process of an ARMA model starting from time series, in addition to the proper choice of the available algorithms and of the procedure to be followed to estimate the parameters among all those available, great attention must be paid on how to properly apply the selected method. Indeed, also in the case considered here, and despite that the selected method—described in Section 3—is based on well-known algorithms, their effectiveness when used together in estimating ARMA models able to accurately describe the sea frequency behaviour strongly depends on the choice of the free parameters.

Even if the parameter selection procedure is fundamental for the achievement of good spectral estimates, no detailed analyses are available in the literature in the field of sea spectral reconstruction. In light of this, this section aims at explaining how to choose the parameters (i.e., the ARMA order $\hat{p}$ and the number of auto-covariance samples, φ) highlighting the limits of both the procedures presented in Section 4 and the effect of the various parameters on the accuracy of the sea spectral estimates. Moreover, it will be shown that also the choice related to the global time length T (i.e., $T=N\Delta_{t}$) of the wave elevation time signal x is a parameter to take into account when applying the estimation procedure since it affects both the accuracy of the spectral estimates and the choice of the free parameters.

In order to analyse the accuracy of the method in estimating the sea spectral behaviour as a function of the free parameters, the index Y has been used. It represents the difference between the reconstructed $S_{x}$, see Equation (21), and the actual JONSWAP spectrum S, see Equation (25), and it is defined as:

(30) $$Y=\sqrt{\frac{\sum_{i=1}^{M}{\left(S_{x}\;\left(\omega_{i}\right)-S\left(\omega_{i}\right)\right)}^{2}}{\sum_{i=1}^{M}{\left(S\left(\omega_{i}\right)\right)}^{2}}\;}$$

The Y value is evaluated in a frequency range between $\omega_{1}$=10^−3^ rad/s and πrad/s, with a frequency resolution of $\Delta_{\omega }$=10^−3^ rad/s. These bounds allow including all the main frequency components of the wave elevation signal.

The following analyses will be thus based on the value of the index Y obtained by applying the method using different values of the free parameters. Before discussing in detail the results of the analyses, it is important to evidence the meaning of Y in terms of goodness of fit of the sea spectral reconstruction.

**Figure 1:**
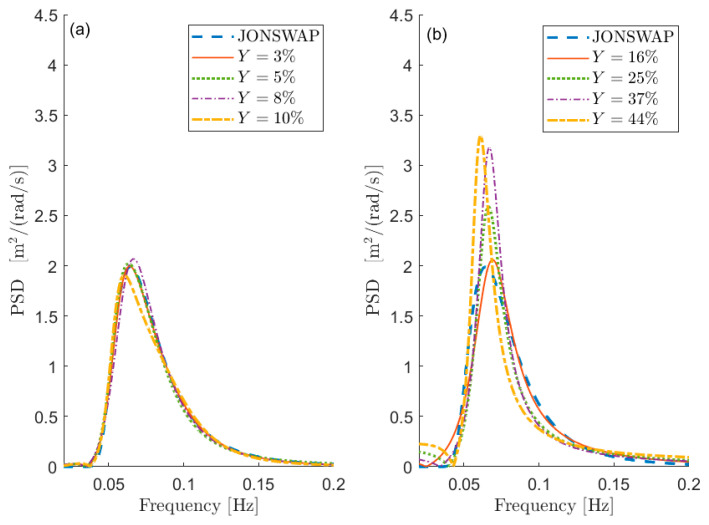
Reconstructed PSD and the corresponding PSD generated with the procedure given in Section 5 (sea state 1 in Table 1) with low (**a**) and larger (**b**) Y values.

To this purpose, Figure 1 depicts some examples of comparisons in terms of PSD between $S_{x}$ and S in the case of test signal 1 in Table 1. The curves are aimed at showing the meaning of a given percentage Y error, and thus the information about the value of the free parameters is not relevant at this stage (i.e., the same Y can be obtained with different combinations of the free parameters). Figure 1a shows the PSD reconstruction associated with low values of Y expressed in percentage (i.e., Y≤0.1) while Figure 1b shows the PSDs associated with larger values of Y (i.e., Y>0.1). Obviously, the lower the value of Y, the better the PSD reconstruction. However, the maximum admissible error on the spectral reconstruction depends on the specific application.

The analyses presented further in this section are related to sea state conditions characterized just by the wind, and thus the considered spectra are mono-modal. The sea parameters considered are those related to type 1 signal in Table 1. However, to show the reliability of the outcomes and of the proposed procedure, also examples related to a different mono-modal sea state and a bi-modal case, where both wind and swell components are present, are discussed in Section 7.

Finally, it is worth mentioning that the proposed estimation procedure does not imply a high computational cost, and that the processing time is of the same order of magnitude of other competing approaches such as Welch’s and Thomson’s methods.

### 6.1. Effect of the Number of the Auto-Covariance Samples Used

The first parameter that has to be defined is the number of equations that can be used to solve the least-square problems involved in the ARMA parameter estimation procedure. Despite the increased AR order $\hat{p}$ may seem the first and most important point to be addressed, it strongly depends on the maximum number of useful information available, given a certain time series x(n). To understand this point, some considerations about the auto-covariance series have to be discussed.

As mentioned previously, the auto-covariance $r_{x}$ of the signal is modelled as a sum of damped exponential components, each corresponding to one pole of the ARMA model (see Equation (13)). Therefore, after some k lags, the value of the estimated auto-covariance becomes low. Considering this aspect and recalling that the auto-covariance is estimated on a limited number of samples (see Equation (12)), two factors must be taken into consideration to judge the reliability of the estimated $r_{x}$ values: the unavoidable variance of the estimated auto-covariance and, in real cases, the presence of electrical noise superimposed to the physical signal provided by the transducer. Both these factors cause a scatter on the auto-covariance values, which becomes more and more significant as k increases, when the oscillations of the auto-covariance become low. Therefore, from the mentioned k-th auto-covariance sample on, the reliability of the auto-covariance curve becomes low and this makes its use for estimating the ARMA model improper. Thus, it is not enough to avoid the use of very large lag values, as suggested in the literature, but it is fundamental to estimate the length of the reliable part of the auto-covariance curve to the aim of estimating the ARMA model. This in turn has consequences on the number of equations that can be written to solve the Prony’s and Shanks’ problems and thus on the maximum order $\hat{p}\;$of the AR model.

One possible approach to select the proper length $\hat{\varphi }$ of $r_{x}$ to use in the estimation procedure, is to refer to the estimate of the auto-covariance of a white noise random process with unitary standard deviation (i.e., $\sigma_{w}=1$), acquired for N samples. The expected value of its normalised auto-covariance ρ(k) (i.e., the auto-covariance divided by its value at the null lag) is null for k > 0 and its standard deviation can be estimated as $1/\sqrt{N}$. Therefore, the auto-covariance samples of a white random process, for k≠0, are assumed to be included in a range of $\pm 3/\sqrt{N}$ with a confidence level of approximately 99%. Considering now the normalised auto-covariance of x (i.e., $\rho_{x}\left(k\right)=r_{x}\left(k\right)/r_{x}\left(0\right)$), the correlation between the signal and its shifted version can be thus considered as not significant if the $\rho_{x}$ curve falls continuously within a range defined as $\pm 3/\sqrt{N}$. Thus, in that region, the low oscillations of $\rho_{x}$ are considered as not useful for the estimation process. This implies that only the first $\hat{\varphi }$ samples of the auto-covariance $r_{x}$ will be used for the estimation of the ARMA model because larger k values show a non-significant correlation level. Thus, $\hat{\varphi }$ indicates the number of auto-covariance lags φ (see Section 3.1) estimated using the $\pm 3/\sqrt{N}$ range, that has to be used to write Equations (14) and (20).

**Figure 2:**
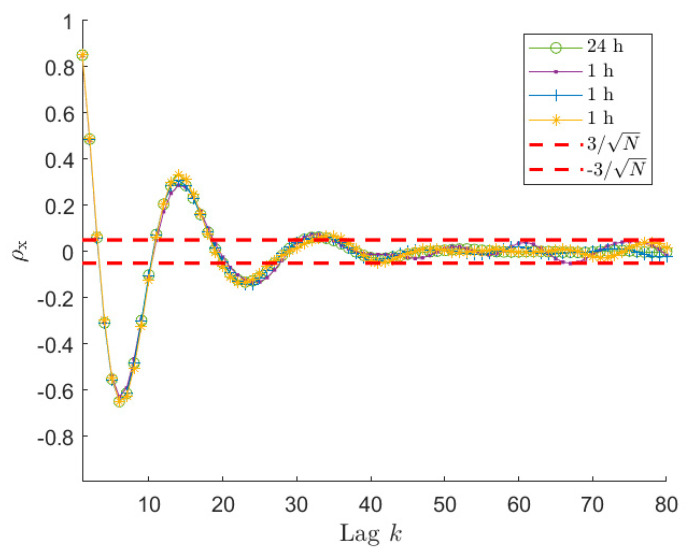
Normalised auto−covariance $\rho_{x}$ curves for a sea state of type 1 in Table 1 for a signal 24 h long and three signals 1 h long, sampled at 1 Hz. The $\pm 3/\sqrt{N}$ range is related to the three 1 h long signals. These three signals have $\hat{\varphi }$ between 34 and 36, depending on the considered time series.

An example of the application of the proposed $\hat{\varphi }$ selection procedure is shown in Figure 2 for signals of type 1 in Table 1, sampled at 1 Hz for 3600 s (i.e., 1 h). In order to give a reference of the theoretical shape of the signal auto-covariance, a sea-state of type 1 has been simulated also considering a time length of 24 h with sampling frequency equal to 1 Hz. Being this auto-covariance computed on a very large number of samples, it can be considered as the reference since, for the amount of lags depicted in the figure, the ratio k/N between the lag order and the total number of samples is considerably low. Figure 2 compares the auto-covariance sequence of three different time-series with the reference curve and with the corresponding $\pm 3/\sqrt{N}$ range. It is possible to notice that the auto-covariance sequences obtained for the three different simulations become significantly different form each other when they are almost contained in the $\pm 3/\sqrt{N}$ range, highlighting the low reliability associated with these data. Instead, except for small differences, the three curves follow the reference auto-covariance in the region of small lags when the $\rho_{x}$ values are not contained in the $\pm 3/\sqrt{N}$ allowing us to identify the reliable part of the auto-covariance sequence. In this way it is possible to identify, for each time series, the $\hat{\varphi }$ number of samples that can be used in the spectral estimation procedure. Obviously, the $\hat{\varphi }$-th sample slightly changes simulation by simulation because of its associated scatter (for the three time series of Figure 1 it is between 34 and 36). Nevertheless, this difference has negligible effects on the quality of the ARMA model identification, because the first and significant oscillations of $\rho_{x}$ are properly described.

**Figure 3:**
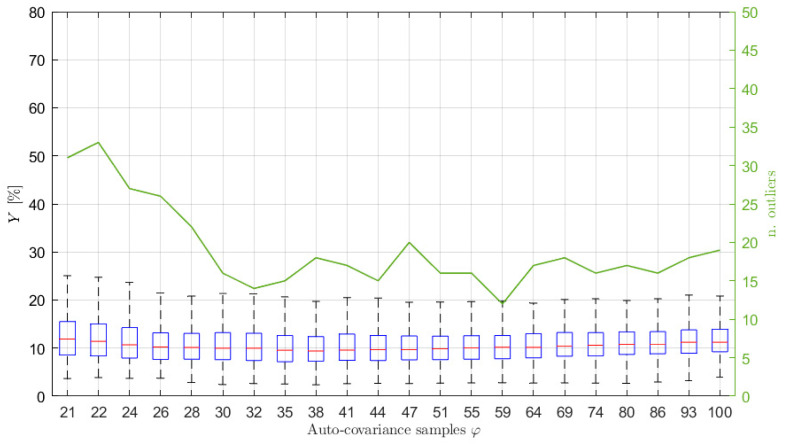
Box plot (left vertical axis) and number of outliers (solid line and right vertical axis) as functions of the number φ of auto−covariance samples used to write Equation (14) for $\hat{p}$ = 10, x 3600s long and sampled at 1 Hz. The typical values of $\hat{\varphi }$ are between 34 and 36.

In order to show the importance of choosing the right number of auto-covariance samples to be considered, and to show the reliability of the proposed selection procedure, several simulations have been carried out, varying the number of the auto-covariance samples used in the estimation process, φ. Particularly, 500 simulations for each selected φ value have been performed. For each of them, the Y index has been evaluated to quantify the accuracy of the estimation procedure. The results are presented in terms of box-plots (showing the Y percentage values of the median, the minimum, the maximum and the 25-th and 75-th percentiles) and of the number of outliers obtained for different φ values, starting from the minimum needed to solve Equation (14) (i.e., $2\hat{p}+1$). Figure 3 shows the results obtained considering signals of type 1 of Table 1, acquired at a sampling frequency of 1 Hz for 1 h. In this case, the increased number of poles $\hat{p}$ has been set equal to 10 (details about the order selection procedure are given in Section 6.2) and q has been set equal to p, without any loss of generality (the same will apply for all the examples further in the paper). As mentioned previously, for this kind of sea wave elevation signal, the typical $\hat{\varphi }$ values are between 34 and 36 (see Figure 2).

Looking at Figure 3 it is interesting to notice that, when φ increases over approximately $\hat{\varphi }$, the value of Y slightly increases (a larger increase occurs for larger φ values, not shown in the figure) because of the use of non-significant values of the auto-covariance sequence. Further details and evidence of this aspect are also given in Section 6.2. In the same way, the use of a number of samples that is too low (i.e., few samples more than those strictly necessary for solving Equation (14)) leads to poor results. Indeed, the left part of Figure 3, if compared to the area close to $\hat{\varphi }≃34$, shows higher values of the median error Y, a wider range between the maximum and the minimum and between the 25-th and 75-th percentiles, as well as a higher number of outliers, evidencing a higher dispersion of the estimation results and a greater variability of the spectral estimates. Therefore, the proposed approach to find $\hat{\varphi }$ shows to be effective since the range of the best φ values in the graph (i.e., the lowest Y values) is found to be close to the $\hat{\varphi }$ values. Moreover, it is noticed that slight changes of $\hat{\varphi }$, such as those deriving from the use of different time series (e.g., $\hat{\varphi }≃34-36$) does not imply considerable changes in the estimation performance, but rather a slight increase (i.e., few lags) with respect to $\hat{\varphi }$ slightly improves the estimates. Thus, a slight increased value of $\hat{\varphi }$ is suggested in most of the cases (see also Section 6.3 for further details).

Therefore, this analysis evidences that it is essential to properly estimate the value of φ to be used in the estimation procedure in order to improve the identification of the ARMA model.

Other examples of the effect on the estimation accuracy of the choice of the number of auto-covariance samples used for the estimation are given in the following sections, where also the effect of the choice of the AR order and the length of the time series are considered.

### 6.2. Choice of the Increased AR Order $\hat{p}$

**Figure 4:**
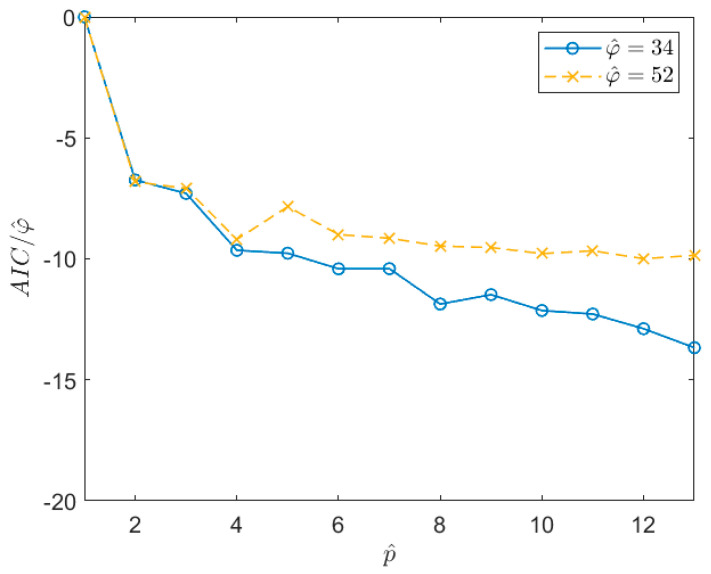
Trend of the AIC index, normalized with respect to the number of the auto−covariance samples used for the estimation procedure, $\hat{\varphi }$. The curves refer to two different time series x(n) of a Type 1 signal in Table 1 sampled at 1  Hz for 1 h.

Once the $\hat{\varphi }$ value is defined, the other parameter that has to be defined is the AR order of the model, p. As explained in Section 4.1, it is convenient to set the pole number to $\hat{p}>p$ and then, once the $\hat{p}$ poles are identified, only the most significant ones in terms of energy are selected. Here, in the most part of the simulations the energy threshold $t_{r}$ was set in order to discard the poles associated with systems whose energy was lower than 10% of the value associated with the system with the highest energy (see Section 4.1 and Section 8 for further details). In Section 4.1, the use of the AIC index is suggested to properly choose the $\hat{p}$ value. Nevertheless, there are cases in which the AIC index (as well as other indexes such as, for example, the Bayesian Information Criterion [41]) does not provide clear indications about how to set the value of $\hat{p}$ (e.g., [31]). In these cases, such as the one considered here (see Figure 4), the value of $\hat{p}$ can be chosen as that allowing us to obtain a first stabilization in the AIC value, even if the AIC is not at its minimum (e.g., it is still slightly decreasing). As an example, Figure 4 shows the trend of the AIC index, normalized with respect to the number of auto-covariance samples used in the estimation procedure, $\hat{\varphi }$. In this case, the signal is of type 1 in Table 1, acquired for 1 h at a sampling frequency of 1 Hz. The two curves are related to two different time series for which a different $\hat{\varphi }$ value was estimated. Looking at the curves, it is evident that the AIC does not provide a clear indication of the optimal $\hat{p}$ value to be used. However, it is possible to see that, after a first drop in correspondence of p almost equal to 4, the curves stabilize. One possibility to set $\hat{p}$ would be to choose a value close to the maximum allowed for the considered time series (i.e., that allowing to write a determined system in Equation (14) for the given $\hat{\varphi }$). However, this reduces the number of equations that can be written to solve the Equations (14) and (20), thus worsening the results of the least square minimisation. Therefore, even if $\hat{p}$ needs to be larger than p, its value must be not too high and a balance between the number of equations that can be used and the increase of the pole number has to be reached. One possible solution is to choose the $\hat{p}$ value inside the stabilized region which guarantees a good number of equations to solve the least square problems and that is, at the same time, far from the first drop of the normalized AIC. Therefore, $\hat{p}$ is set to a value lower than the mentioned maximum, taking care not to move too close to the stabilisation point. In this case, a good balance is a $\hat{p}$ value of about 10. Of course, this choice is subjective (e.g., the value could be 8 or 12 as well); however, this does not modify the obtained results significantly.

**Figure 5:**
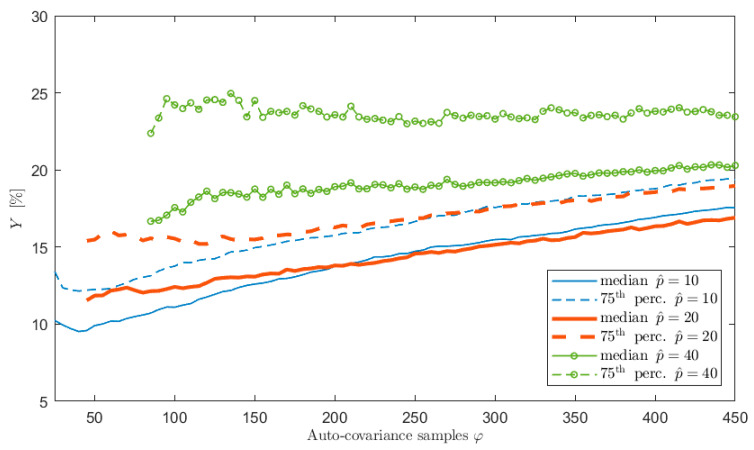
Trend of the median and 75-th percentile values of Y as functions of the number φ of auto−covariance samples used to write Equation (14) for $\hat{p}$ = 10, 20 and 40. x is 3600 s long and sampled at 1 Hz.

To show the effect of the number of poles on the accuracy of the spectral estimates as a function of the number of the auto-covariance samples φ, many simulations were carried out on different wave elevation signals where both the sea state conditions and the length of the time histories were changed. For sake of clarity, and coherently with the test case study of the previous section (so that a straight comparison can be carried out), the results related to 1 h signals of type 1, sampled at 1 Hz, are shown. The considered $\hat{p}$ range in this case was between 10 and 40 poles. The value of the maximum number of poles was set equal to 40 in the simulations to also test values very far from the maximum achievable with the usual $\hat{\varphi }$ in order to see its effect on the accuracy of the PSD estimates. The results are summarized in Figure 5, which shows a comparison in terms of accuracy of the spectral estimates, measured by the index Y, when the increased order of the poles is $\hat{p}\;$= 10, 20 and 40. In the figure, the trends of the median and the 75-th percentile values of Y associated to each $\hat{p}$ value are shown as functions of the value of φ. Comparing the curves in the figure, it is evident that, at a given value of φ, the value of Y is lower for the lowest value of $\hat{p}$ and increasing $\hat{p}$ makes the identification worse (higher values of Y), with the only exception of the right part of the plot where the curves related to $\hat{p\;}$ = 10 and 20 merge. Nevertheless, this part of the plot is related to φ values providing results that are not satisfactory.

The use of a lower level of overestimation of p proves to provide better results because of two main reasons: (i) the use of high values of $\hat{p}$ obliges to employ auto-covariance samples in the lag range where the auto-covariance is low and (ii) for a given value of φ, the number of equations used to solve the least square problems of Equations (14) and (20) is larger for lower values of $\hat{p}$.

Finally, it is worth highlighting that, as already shown in Section 6.1, the use of a number of auto-covariance samples higher than $\hat{\varphi }$ can significantly worsen the achievable performance. Indeed, the curves in Figure 5 show an increase of the Y index for high φ values.

### 6.3. Effect of the Length of the Time Series

**Figure 6:**
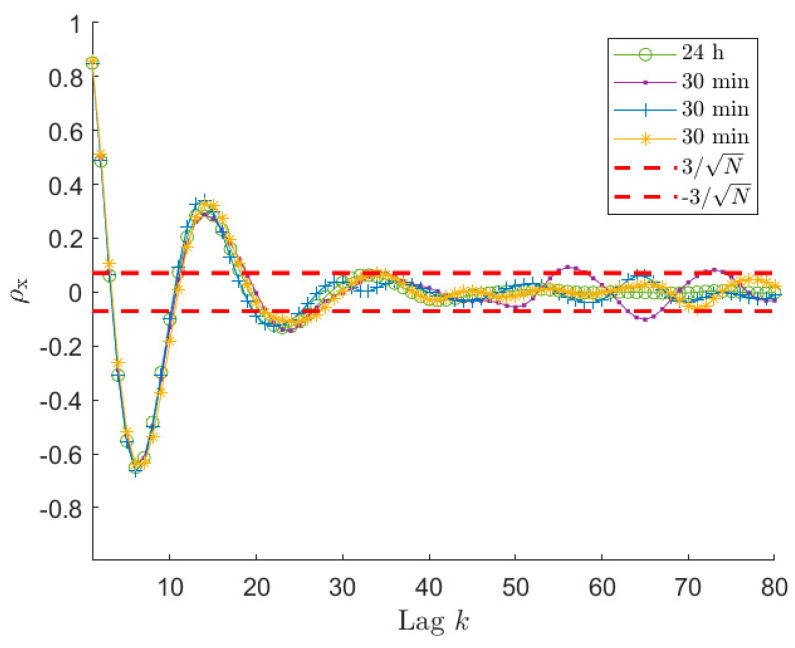
Normalised auto−covariance $\rho_{x}$ curves for a signal 24 h long and three signals 30 min long. The $\pm 3/\sqrt{N}$ range is related to the three 30 min long signals. These three signals have $\hat{\varphi }$ between 25 and 27, depending on the time series.

**Figure 7:**
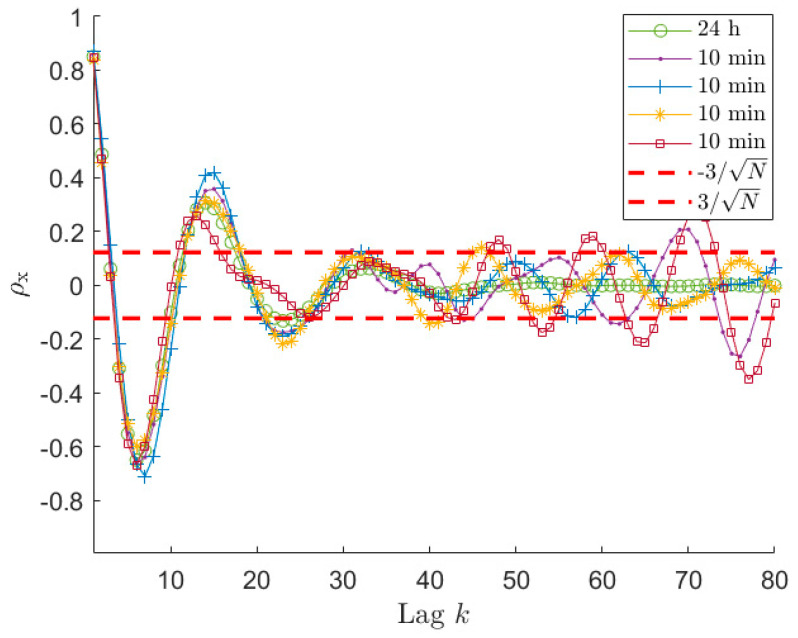
Normalised auto−covariance $\rho_{x}$ curves for a signal 24 h long and four signals 10 min long. The $\pm 3/\sqrt{N}$ range is related to the four 10 min long signals. These four signals have $\hat{\varphi }$ between 17 and 25, depending on the time series.

So far, all the examples used to support the presented analyses were related to long time histories, in particular 1 h time series were considered. Despite the proposed procedure and all the results are valid whichever signal is taken into account, it is worth paying attention to the problems that can arise when short-time signals are considered and showing the results that can be achieved in terms of accuracy of the estimated sea PSD. Indeed, in these cases, problems can arise, making the use of the proposed procedure more cumbersome. To this purpose, sea wave elevation signals of 30 and 10 min are analysed, using a sea state of type 1 of Table 1, also to allow for a comparison with the already presented results. The first point that has to be analysed is the auto-covariance behaviour of the time series. Figure 6 and Figure 7 present normalised auto-covariances $\rho_{x}$ achieved using x signals of 30 min and 10 min length, respectively. As mentioned in Section 6.1, the auto-covariance plot allows us to select the reliable portion of the samples that have to be used in the estimation procedure, since this allows for more accurate PSD estimates. However, the identification of the $\hat{\varphi }$ value using the $\pm 3/\sqrt{N}$ can be not so straightforward if the length of the time series decreases too much. Indeed, looking at Figure 6, it is possible to notice that $\rho_{x}$ for the 30 min signal, after entering the $\pm 3/\sqrt{N}$ band, starts oscillating and the amplitude of these oscillations increases as the lag increases. It can be also seen that one of the curves in Figure 6 overcomes the upper threshold due to the large scatter. Comparing Figure 2, Figure 3, Figure 4, Figure 5 and Figure 6, it appears that this behaviour is instead not evident for the 1 h time series. Actually, also in the case of long time series (e.g., 1 h long) the behaviour is similar; however, it is related to higher lag values. This happens because, decreasing the time length (e.g., from 1 h to 30 min), even if the $\pm 3/\sqrt{N}$ bounds become larger, at the same lag value the scatter associated with the auto-covariance of the short signal, is larger compared to the case of long signals since the considered lag is closer to the tail of the auto-covariance (i.e., higher k/N value).

**Figure 8:**
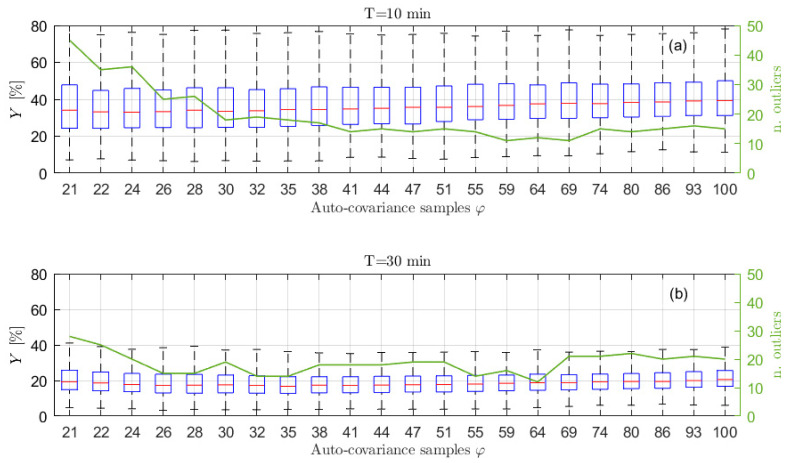
Box plot (left vertical axis) and number of outliers (solid line and right vertical axis) as functions of the number φ of auto−covariance samples used to write Equation (14) obtained using $\hat{p}$ = 10 and signals of type 1 in Table 1, sampled at 1 Hz for 600 s (**a**) and 1800 s (**b**). The typical values of $\hat{\varphi }$ are between 25 and 27 for 30 min time series and between 17 and 25 for 10 min.

Therefore, it is evident that, lowering the total number of samples, the auto-covariance shows a larger scatter for a fixed lag value. This can become a problem in identifying the significant portion of $\rho_{x}$. However, the important point for the application of the method proposed to choose φ is to have the auto-covariance series within the $\pm 3/\sqrt{N}$ for a significant number of lags in order to recognize when the auto-covariance becomes non-significant for the purpose of the ARMA identification. Looking at Figure 2 and Figure 6, it is easy to notice that this is verified for time signals of 1 h and 30 min. Therefore, the procedure of Section 6.1 allows us to identify $\hat{\varphi }$ values related to portions of the auto-covariance series that are close to the reference auto-covariance curve, implying employing a part of $r_{x}$ that is significant (i.e., $\hat{\varphi }≃25-27$ for 30 min signals, see Figure 6). However, in the two cases, the identified value of $\hat{\varphi }$ is different and in particular decreases from 1 h to 30 min signals (i.e., from $\hat{\varphi }≃34-36$ to $\hat{\varphi }≃25-27$) because of the larger value of $\pm 3/\sqrt{N}$. This helps in avoiding using portions of the $r_{x}$ that are too affected by its scatter, but in turn it limits the number of available points, and this has an effect on the achievable identification performance, as described in Section 6.1. To show this effect and the accuracy of the PSD estimates achievable with time histories of 30 min and 10 min, Figure 8 presents the results in terms of box plots and number of outliers, as those of Figure 3 for series 1 h long. Considering signals 30 min long, from Figure 8b it can be noticed that, as in the previous case, the best results are achieved for a φ value close to $\hat{\varphi }$. Moreover, even if the quality of the reconstruction is lower reducing the time length (see Figure 3 and Figure 8b), as expected, it is still satisfactory, which in turn means that 30 min is an acceptable time length for a proper ARMA identification.

If the length of the time signal is further decreased, for example to 10 min, the problems discussed previously become more and more evident. Considering Figure 7, which shows the auto-covariance for signals 10 min long, it is clear that it becomes difficult to clearly identify a lag range after the first oscillations where the curve remains within the $\pm 3/\sqrt{N}$ range. Moreover, the four auto-covariance series are different even for small lag values (e.g., at lag 15), because of the lower ratio between the lag value and the total amount of samples of the original signal, meaning that the usable portion of the auto-covariance curve is strongly decreased. Therefore, sometimes, it can occur that the criterium proposed to find $\hat{\varphi }$ leads to a very small number of usable lag values. In these cases, there are various possibilities to manage the situation. One possibility is to use a small value for $\hat{p}$. This case is treated in Section 6.4, as well as the possibility to use an increased sampling frequency for the time series x. The other possibility is to use a $\hat{p}$ values that is usually fine for longer time series (e.g., $\hat{p}$ = 10) and accepting to increase φ to a value allowing to write a determined (or slightly overdetermined) system for the solution of the Prony’s problem. This case is treated in this section in order to compare the identification results when the time length of x is changed. For example, Figure 7 shows that, in the case of 10 min signals in some cases the $\hat{\varphi }$ lag is around 24 while for some others the value of $\hat{\varphi }$ can be around the 17-th lag. In these latter cases, $\hat{\varphi \;}$ is not enough for writing all the required equations in Equation (14) with $\hat{p}=10$. Therefore, the number of the auto-covariance points that has to be considered must be increased at least up to 21. In order to show the effect of these problems on the accuracy of the estimated PSD, Figure 8a presents the box plot for the signals 10 min long with $\hat{p}=10$. Since the observed phenomenon is always the same (i.e., sea state of type 1), the choice of maintaining the $\hat{p}$ value equal to the previous cases is reasonable. Figure 8a, shows that increasing φ over 21 (i.e., the number of auto-covariance samples to be used for having as many equations as the number of unknowns in Equation (14)), provides a very slight decrease of the value Y and the number of outliers. Conversely, if the number of the used auto-covariance samples increases over about 24, the value Y starts increasing due to the fact that the added auto-covariance samples are already unreliable. Indeed, even if using 26 lag samples (as an example) means to employ few unreliable auto-covariance samples, their number is not negligible compared to the total number of auto-covariance samples used for the identification, thus turning out in a worsening of the procedure results. Therefore, in the cases of short time series, it is not convenient to slightly increase $\hat{\varphi }$ value, as instead occurred in the case of long-time signals. Moreover, Figure 8a shows that the Y results for 10 min are far from those achievable with either 30 or 60 min for all the possible φ values.

As mentioned, other possible ways to deal with short-time series are: the use of a lower number of poles $\hat{p}$ and the increase the sampling frequency. These methods, explained in the next section, will also allow to slightly improve the estimation accuracy.

### 6.4. Short-Time Series: Effect of the Number of Poles and the Sampling Frequency

**Figure 9:**
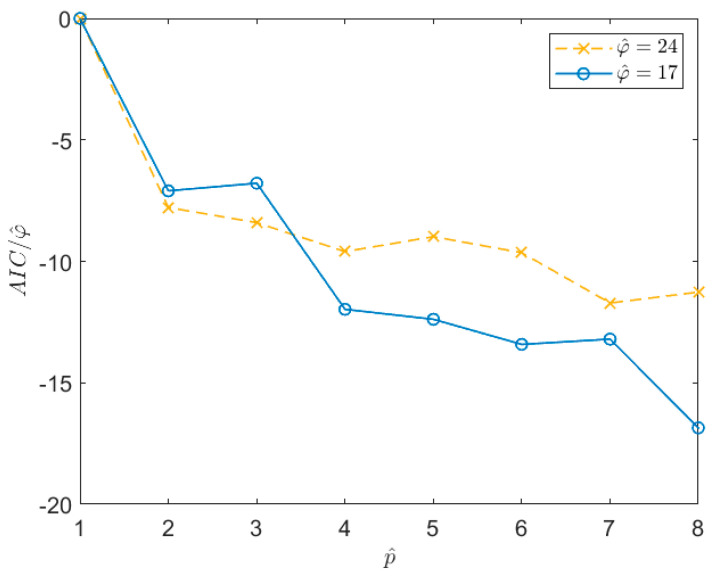
Trend of the AIC index, normalized with respect to the number of the auto−covariance samples used for the estimation procedure. The curves refer to two different time series of Type 1 signals in Table 1 sampled at 1 Hz for 10 min.

**Figure 10:**
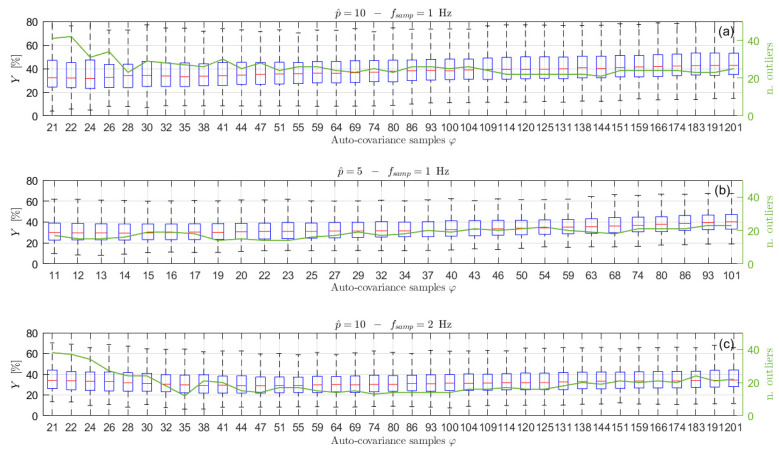
Box plot (left vertical axis) and number of outliers (solid line and right vertical axis) as functions of the number φ of auto−covariance samples used to write Equation (14) for x 600 s long with $\hat{p}$ = 10 and $f_{samp}=1$ Hz (**a**) $\hat{p}$ = 5 and $f_{samp}=1$ Hz (**b**) and = 10 and $f_{samp}=2$ Hz (**c**).

The analyses of the previous section showed the problems that can arise when dealing with short-time series, due to the scatter of the auto-covariance samples that occur even for low lag values. One possibility to try to improve the achievable estimates is to reduce the increased order $\hat{p}$ of the AR problem. This would allow increasing the overdetermination level of the least square problems of Equations (14) and (20) and thus to improve the accuracy of the estimates. However, the increase of the poles used to solve the first step of the estimation procedure will be lower than in the previous case (i.e., the difference between $\hat{p}$ and p will be lower). This choice can have consequences in the pole-selection procedure based on the energetic criterion and also implies the risk of choosing a $\hat{p}$ value too close or even lower than p. In order to evaluate the effect of the order reduction on the estimates, a new lower $\hat{p}\;$value has been selected for the 10 min long time series, following the criterion described in Section 6.2. The trend of the normalized AIC index is shown in Figure 9 for two different 10 min long time series. In this case the $\hat{p}$ value must be close to the first drop of the AIC value in order to be able to solve in the least square sense the minimisation problem of Equation (14). Therefore, Figure 9 suggests in this case a value of $\hat{p}$ of about 5, though, as in the previous case, its interpretation is not straightforward. With this value of $\hat{p}$, new simulations have been carried out to evaluate the accuracy of the obtained sea PSD estimates, which are shown in Figure 10b. In this case, in order to allow for a straight comparison with the results related to the case where the sampling frequency is increased (see further in this section), also some noise has been added to the wave elevation signal. The signal-to-noise ratio has been set equal to 30 dB (assuming a noise having flat power spectrum). Before analysing the results, it is worth mentioning that the effect of an increased noise level does not affect significantly the results shown for 30 min and 1 h time series as well as for 10 min, as mentioned further in this section, mainly thanks to the use of auto-covariance series in the estimation process and to the least square solution of the problems involved in the proposed procedure. Comparing Figure 10a where $\hat{p}=10$ to Figure 10b where $\hat{p}=5$, and the signal-to-noise-ratio is the same, it is evident that the results obtained using $\hat{p}=5$ shows an improvement of the accuracy of the spectral reconstruction of few percentage points (i.e., about 5%), though the achieved results do not reach the accuracy values achievable with longer time series (see Figure 3 and Figure 8b).

Another possible way to improve the results achievable with short-time series is to slightly increase the sampling frequency of the signal. In order to verify if this could provide benefits, simulations have been carried out increasing the sampling frequency of the original time sequence x. When the sampling frequency $f_{samp}$ is increased, different factors must be considered:

- in real cases, electrical noise is superimposed to the physical signal in the time sequence *x*, and an increase of the sampling frequency turns into an increase of the noise power. This implies a larger scatter associated to the auto-covariance sequence, especially when its value is low. Therefore, this worsens the ARMA identification;
- on the other hand, a larger number of samples can be used to write Equation (14), thus improving the least square solution, because, for the same lag value in the time domain (i.e., for the same $k\Delta_{t}$), a larger number of lag samples is obtained due the smaller value of $\Delta_{t}$;
- given the spectral content of the wave elevation signals (i.e., far below 1 Hz) and the values of sampling frequency considered (i.e., 1 Hz), the expected improvement of the auto-covariance estimates provided by an increase of the sampling frequency is low. Indeed, according to [42], only a slight decrease of the auto-covariance scatter is obtained increasing $f_{samp}$ from 1 Hz.

According to the above points, no large improvements can be obtained by increasing $f_{samp}$. Simulations using $f_{samp}$ = 2 Hz for the case of 10 min long signals, $\hat{p}=10$ and noise added (signal-to-noise ratio equal to 30 dB for $f_{samp}$ = 1 Hz and to approximately 27 dB for $f_{samp}$ = 2 Hz, assuming noise with a flat power spectrum) confirmed this expectation. It is noticed that is straightforward to adapt the procedure described in Section 3 to non-unitary sampling frequency. Comparing Figure 10a,c, where only the sampling frequency changes, the best performance obtained for the highest sampling frequency is slightly better than that obtained with $f_{samp}=1$. The main benefit provided by employing $f_{samp}$ = 2 Hz is related to the fact that changes in the number of lags used to write Equation (14) generate less changes of the results (see the right part of plot (c)), compared to the case of 1 Hz. Furthermore, as mentioned, comparing Figure 8a and Figure 10a, it is evident that the noise does not cause significant worsening of the ARMA identification because the two figures show similar Y results, regardless of the presence of noise. Moreover, comparing Figure 10b,c, it is clear that both the proposed strategies (i.e., decrease of $\hat{p}$ and increase of $f_{samp}$) provide the same slight performance improvement; thus, neither the use of one nor the other is preferred. Finally, it is worth mentioning that use of the two strategies together leads to further slight improvements (i.e., about 1 percentage point).

Finally, to summarize, this analysis evidenced that a decrease of the time length of x (e.g., under 20 min) always implies a worsening of the model identification. Furthermore, for signals x with short duration, the variance associated to $\rho_{x}$ is significant compared to the range $\pm 3/\sqrt{N}$ even at low order lag values, thus making difficult the use of the proposed criterion for the choice of $\hat{\varphi }$. Therefore, the two proposed strategies can be applied to slightly improve the estimation accuracy, that are to choose a low $\hat{p}$ value or to slightly increase the sampling frequency.

## 7. PSD Estimation Results for Different Sea Conditions

**Figure 11:**
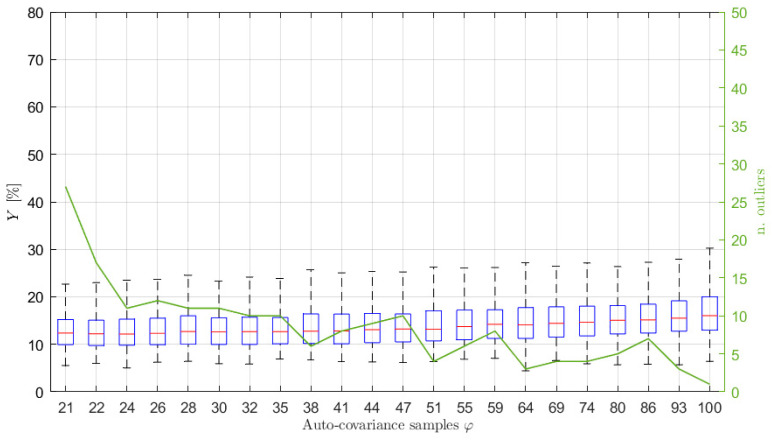
Box plot (left vertical axis) and number of outliers (solid line and right vertical axis) as functions of the number φ of auto−covariance samples used to write Equation (14) for sea state 2 in Table 1, $\hat{p}$ = 10, x 3600 s long and sampled at 1 Hz. The typical values of $\hat{\varphi }$ are between 23 and 27.

**Figure 12:**
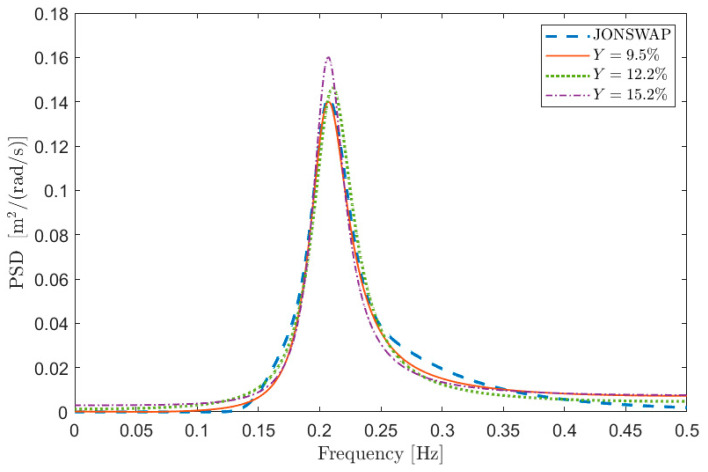
Reconstructed PSD and the corresponding PSD generated with the procedure given in Section 5 (sea state 2 in Table 1) for different Y values.

**Figure 13:**
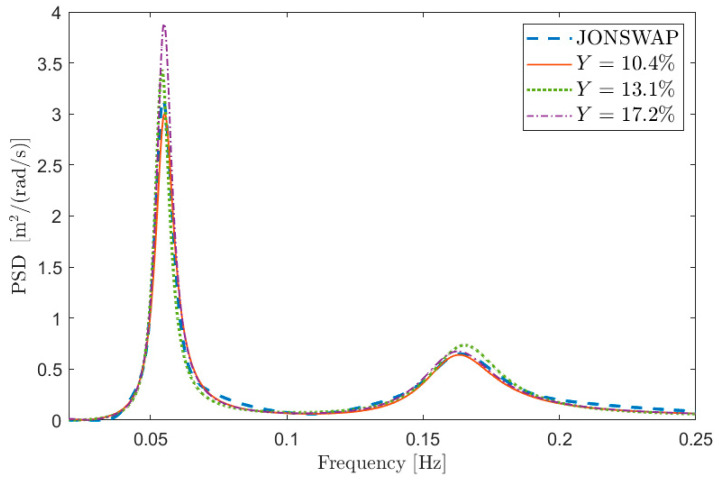
Reconstructed PSD and the corresponding PSD generated with the procedure given in Section 5 (sea state 3 in Table 1) for different Y values.

Finally, to show the reliability of the proposed procedure, some results are shown for different mono-modal and bi-modal sea state conditions. In particular, sea states 2 and 3 of Table 1 have been considered and signals of 3600 s long, with $\hat{p}$ = 10 and with $f_{samp}$ = 1 Hz have been simulated using the procedure of Section 5. The box plot of Y is shown in Figure 11 for sea state 2. It is evident that the performance of the reconstruction is not far from that obtained for sea state 1 using the same simulation parameters (see Figure 3). Some reconstructed PSDs compared to the reference one are also provided in Figure 12. The shown PSDs are related to Y values close to the median, 25-th percentile and 75-th percentile values and are found using a number of auto-covariance samples equal to $\hat{\varphi }$. Finally, some identifications have been carried out for sea state 3 (bi-modal sea state) using again the number of samples obtained applying the range $\pm 3/\sqrt{N}$ for the model identification process. The median value of Y resulted equal to approximately 13%, while the 25-th and 75-th percentiles to approximately 10% and 17%, respectively, thus evidencing a good match between the original and reconstructed PSD. Some reconstructed PSDs are compared to the reference one in Figure 13, for Y values close to the median, 25-th percentile and 75-th percentile values. Therefore, the proposed method and the suggested procedures to set the parameters involved in the analysis show good results even when different sea states are considered. Indeed, the method allows for achieving good sea PSD reconstructions even though, especially in the case of bi-modal sea state, a deeper analysis needs to be carried out and just a first analysis is presented.

## 8. Guidelines

The discussions presented in the previous sections showed the effect of the parameters involved in the ARMA model estimation process on the accuracy and on the effectiveness of the proposed approach when the frequency behaviour of sea wave elevation has to be estimated. The main parameters that have to be set are the number of poles of the increased order AR model, $\hat{p}$, and the number of auto-covariance samples that can be used in the estimation process, φ. These two parameters are shown to be strictly related and to significantly influence the effectiveness of the procedure. The analyses carried out allow for defining guidelines that can be followed to set the $\hat{p}$ and φ value. They can be chosen as follows:

- the value of φ can be estimated by plotting the normalised auto-covariance $\rho_{x}$ of the considered time series x(n) and finding the lag for which its oscillations remain within a range $\pm 3/\sqrt{N}$. It is also noticed that simulations suggest to increase of some samples the φ value obtained with the range $\pm 3/\sqrt{N}$ because this slightly improves the spectral reconstruction. This latter consideration does not hold for short time series;
- the value of $\hat{p}$ has to be overestimated compared to p, that is unknown, however, it must not be increased excessively not to worsen the ARMA identification. To select a suitable value of $\hat{p}$, the AIC index can be used and $\hat{p}$ can be chosen as the value which allows to obtain a stabilization in the AIC value after the first drop, even if the AIC is not at its minimum, and at the same time to solve the Prony’s and Shanks’ problems in the least square sense. The simulations carried out showed that when the sea state is mono-modal, a reasonable value of $\hat{p}$ can be about 10. Higher values showed to be useless. For short-time series, this value can be decreased to 5 allowing the use of a reasonable number of auto-covariance samples in the minimisation procedure. For bi-modal sea, few analyses on long time histories were carried out so far, which showed similar results and suggested similar values of $\hat{p}$. However, the procedure described in Section 5 allows for simulating different sea states, making it possible to have a more detailed estimate of the possible value of $\hat{p}$ in case of given sea states.
- A slight increase of the sampling frequency provides little improvement in the estimation accuracy when short time series are used.
- A final remark is related to the energy threshold $t_{r}$ used for the selection of the final AR order of the ARMA model, starting from an increased AR order model, $\hat{p}$. In these analyses, it was set equal to an energy level at least equal to a given percentage $P_{e}$ of the energy of the subsystem with the highest energy (see Equation (24)). Usually, in these analyses, $P_{e}$ was set to 10%. Therefore, the discarded poles were those associated to systems whose energy was lower than 10% of the value associated to the system with the highest energy. For mono-modal sea states, this choice often led to a final AR order equal to 4. Nevertheless, it was found that lowering $P_{e}\;$to 5% does not generate significant changes of the results for mono-modal sea state, while it provides benefits in case of bi-modal sea state.

**Figure 14:**
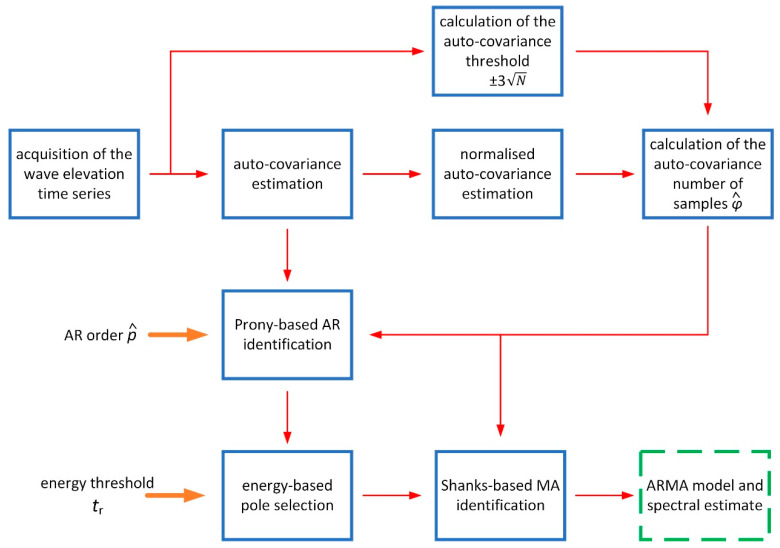
Workflow of the ARMA model estimation procedure. Thick orange arrows indicate user inputs, blue boxes the various steps of the procedure, thin red arrows their outputs and the green dashed box the final estimation result.

Finally, in light of these considerations, the whole spectral estimation procedure can be summarised by the workflow depicted in Figure 14. In case of applications aimed at continuous sea state monitoring, the values of the input parameters $\hat{p}$ and $t_{r}$ can be periodically checked and updated, following the abovementioned procedure.

## 9. Conclusions

The paper proposes a procedure to estimate an ARMA model for the purpose of the spectral reconstruction of wave elevation time series and to properly set the involved parameters. Indeed, its main focus was on an analysis aiming at comprehending the performance in terms of reconstruction accuracy when the identified ARMA model is used. Specific focus has been given to three different parameters that must be set before carrying out the identification and the spectral reconstruction: the global length of the signal x, the number of poles $\hat{p}$ to be initially assigned to the model to be estimated, and the number of auto-covariance samples $\hat{\varphi }$ to be employed for the identification procedure. The paper proposes some guidelines to properly set these parameters in order to obtain the best possible accuracy for a given time series.

Moreover, the analysis of Section 6 has shown that the total time length T of the signal x is a critical parameter for a proper frequency reconstruction of the sea state. Indeed, for T lower than 1800 s (i.e., 30 min), that corresponds to N equal to 1800 for $f_{samp}$ = 1 Hz, the quality of the reconstruction worsens significantly even when optimal parameters are used. Conversely, for larger T values the results are satisfactory, and the strategy proposed to find the number of auto-covariance samples and the AR order works properly. When T is low, an increase of the sampling frequency can lead to some benefits, even if the reconstruction performance remains far from those achieved increasing T.

The main part of the analysis has been focused to mono-modal sea states, even if preliminary analyses show good performances also on bi-modal sea states. A deeper insight will be necessary in this case in order to understand how to take into consideration the different decay rates of the different components of the auto-covariance function. Moreover, the authors are currently studying some approaches to improve the spectral reconstruction through ARMA models in case of signals of short duration. The paper demonstrated the potentiality of the approach and the feasibility of its use in the field of sea state analysis when the parameters involved in the estimation process are properly chosen. This justifies future studies, currently in progress, on the comparison of the present approach with other competing spectral estimation methods, presently investigated for the purpose of sea spectral reconstruction.

## Figures and Tables

**Table 1:** Sea state parameters used for the wave elevation simulations.

Test Signal Type	Wind				Swell			
	$H_{s}$ [m]	$T_{m}$ [s]	$T_{p}$ [s]	γ [-]	$H_{s}$ [m]	$T_{m}$ [s]	$T_{p}$ [s]	γ [-]
1	3.00	12.00	15.51	1.00	-	-	-	-
2	1.00	4.00	4.82	3.00	-	-	-	-
3	2.00	5.00	6.11	2.50	2.00	16.00	18.32	6.50

## Data Availability

The data presented in this study are available on request from the corresponding author.

## References

[1] Gaglione S., Piscopo V., Scamardella A. (2016). The overall motion induced interruptions as operability criterion for fishing vessels. J. Mar. Sci. Technol..

[2] Scamardella A., Piscopo V. (2014). Passenger ship seakeeping optimization by the Overall Motion Sickness Incidence. Ocean Eng..

[3] Böhm M., Kowalski M. (2020). Fatigue life estimation of explosive cladded transition joints with the use of the spectral method for the case of a random sea state. Mar. Struct..

[4] IMO (2007). Revised Guidance to the Master for Avoiding Dangerous Situations in Following and Quartering Seas.

[5] Nielsen U.D. (2017). A concise account of techniques available for shipboard sea state estimation. Ocean Eng..

[6] Montazeri N., Nielsen U.D., Juncher Jensen J. (2016). Estimation of wind sea and swell using shipboard measurements—A refined parametric modelling approach. Appl. Ocean Res..

[7] Iseki T., Ohtsu K. (2000). Bayesian estimation of directional wave spectra based on ship motions. Control Eng. Pract..

[8] Piscopo V., Scamardella A., Gaglione S. (2020). A new wave spectrum resembling procedure based on ship motion analysis. Ocean Eng..

[9] Pascoal R., Guedes Soares C., Sørensen A.J. (2007). Ocean wave spectral estimation using vessel wave frequency motions. J. Offshore Mech. Arct. Eng..

[10] Brandt A. (2011). Noise and Vibration Analysis—Signal Analysis and Experimental Procedures.

[11] Percival D.B., Walden A.T. (2020). Spectral Analysis for Univariate Time Series.

[12] Rossi G.B., Crenna F., Piscopo V., Scamardella A. (2020). Comparison of Spectrum Estimation Methods for the Accurate Evaluation of Sea State Parameters. Sensors.

[13] Rossi G.B., Crenna F., Berardengo M., Piscopo V., Scamardella A. Data processing for the accurate evaluation of combined wind sea and swell spectra. Proceedings of the 2020 IMEKO TC-19 International Workshop on Metrology for the Sea.

[14] Rossi G.B., Crenna F., Berardengo M., Piscopo V., Scamardella A. (2021). Investigation on Spectrum Estimation Methods for Bimodal Sea State Conditions. Sensors.

[15] Ge M., Kerrigan E.C. Short-term ocean wave forecasting using an autoregressive moving average model. Proceedings of the 2016 UKACC 11th International Conference on Control.

[16] Spanos P.D., Zeldin B.A. (1996). Efficient Iterative Arma Approximation of Multivariate Random Processes for Structural Dynamics Applications. Earthq. Eng. Struct. Dyn..

[17] Spanos P.T.D. (1983). ARMA algorithms for ocean wave modeling. J. Energy Resour. Technol. Trans. ASME.

[18] Mandal S., Witz J.A., Lyons G.J. (1992). Reduced order ARMA spectral estimation of ocean waves. Appl. Ocean Res..

[19] Kohn A.F., Akay M. (2006). Autocorrelation and Cross-Correlation Methods. Wiley Encyclopedia of Biomedical Engineering.

[20] Gersch W. (1970). Estimation of the autoregressive parameters of a mixed autoregressive moving-average time series. IEEE Trans. Autom. Control.

[21] Hu S., Wu S.M. (1989). Prony estimation of AR parameters of an ARMA time series. Mech. Syst. Signal Process..

[22] Michelini R.C., Rossi G.B. The PSD measurement of ARMA processes correlation by exponential modelling. Proceedings of the IASTED International Symposium on Applied Signal Processing and Digital Filtering.

[23] Michelini R.C., Rossi G.B. Test validation of the PSD measurement for exponentially correlated processes. Proceedings of the IASTED International Symposium on Applied Signal Processing and Digital Filtering.

[24] Shanks J.L. (1967). Recursion Filters for Digital Processing. Geophysics.

[25] Pandit S.M. (1991). Modal and Spectrum Analysis: Data Dependent Systems in State Space.

[26] James III G.H., Carne T.G., Lauffer J.P. (1995). The Natural Excitation Technique (NExT) for Modal Parameter Extraction from Operating Structures. Int. J. Anal. Exp. Modal Anal..

[27] Ewins D.J. (2009). Modal Testing: Theory, Practice and Application.

[28] Hasan M.K., Fattah S.A., Khan M.R. (2003). Identification of noisy AR systems using damped sinusoidal model of autocorrelation function. IEEE Signal Process. Lett..

[29] Cadzow J.A. (1980). High Performance Spectral Estimation—A New ARMA Method. IEEE Trans. Acoust..

[30] Kay S.M. (1980). A new ARMA spectral estimator. IEEE Trans. Acoust. Speech Signal Process..

[31] Friedlander B., Porat B. (1984). The Modified Yule–Walker Method of ARMA Spectral Estimation. IEEE Trans. Aerosp. Electron. Syst..

[32] Akaike H., Nakagawa T. (1988). Statistical Analysis and Control of Dynamic Systems.

[33] McQuarrie A., Shumway R., Tsai C.L. (1997). The model selection criterion AICu. Stat. Probab. Lett..

[34] Eichstädt S., Elster C., Esward T.J., Hessling J.P. (2010). Deconvolution filters for the analysis of dynamic measurement processes: A tutorial. Metrologia.

[35] Box G.E.P., Jenkins G.M., Reinsel G.C., Ljung G.M. (2016). Time Series Analysis—Forecasting and Control.

[36] Anderson O.D. (1977). The Box-Jenkins Approach To Time Series Analysis. RAIRO Rech. Oper..

[37] (2014). DET NORSKE VERITAS, Recommended Practice DNV-RP-C205—Environmental Conditions and Environmental Loads. https://rules.dnv.com/docs/pdf/dnvpm/codes/docs/2014-04/RP-C205.pdf.

[38] Ewans K.C., Bitner-Gregersen E.M., Soares C.G. (2006). Estimation of wind-sea and swell components in a bimodal sea state. J. Offshore Mech. Arct. Eng..

[39] Pierson W.J., Moskowitz L. (1964). A proposed spectral form for fully developed wind seas based on the similarity theory of S. A. Kitaigorodskii. J. Geophys. Res..

[40] Chakrabarti S.K. (2005). Handbook of Offshore Engineering.

[41] Ljung L. (1999). System Identification: Theory for the User.

[42] Kay S.M. (1981). The Effect of Sampling Rate on Autocorrelation Estimation. IEEE Trans. Acoust..

